# Robust, high brightness, degenerate entangled photon source at room temperature

**DOI:** 10.1038/s41598-017-12709-5

**Published:** 2017-10-03

**Authors:** M. V. Jabir, G. K. Samanta

**Affiliations:** Photonic Sciences Lab., Physical Research Laboratory, Navarangpura, Ahmedabad, 380009 Gujarat India

## Abstract

We report on a compact, simple and robust high brightness entangled photon source at room temperature. Based on a 30-mm-long periodically-poled potassium titanyl phosphate crystal, the source produces non-collinear, type-0, phase-matched, degenerate photons at 810 nm with spectral brightness as high as ~0.41 ± 0.02 (~0.025 ± 0.02) MHz/mW/nm for multi (single) mode fiber coupling. So far, this is the highest number of degenerate photons generated using a continuous-wave laser pumped bulk crystal and detected using multimode fiber. We have studied the dependence of pump focusing on the brightness of the generated photons collected using both multimode, and single mode fibers. For a fixed pump power and crystal parameters, the SPDC source has an optimum pump waist radius producing maximum number of paired photons. Combining the crystal in a novel system architecture comprised with Sagnac interferometer and polarizing optical elements, the source produces polarization entangled photon states with high spectral brightness. Even in the absence of any phase compensation, the entangled photon states detected using single mode fiber have a Bell’s parameter, S = 2.63 ± 0.02, violating the Bell’s inequality by nearly 32 standard deviations and fidelity of 0.975. The compact footprint, robust design, and room temperature operation, make our source ideal for various quantum communication experiments.

## Introduction

Entangled photon sources, a basic ingredient for many quantum optical experiments, are of paramount importance not only for the fundamental research^1^ but also for a variety of applications in real world quantum communications^2^ and quantum computing^3^. However, the realization of next generation envisaged projects towards the implementation of world-wide quantum network through ground-to-satellite and or inter-satellite links^4,5^ require development of compact and robust entangled photon sources with high brightness, and entanglement purity. Over decades, a variety of schemes have been proposed and implemented^6,7^ for entangled photons, however, the polarization entangled photon sources realized through the spontaneous parametric down-conversion (SPDC) in second order, (χ^2^), bulk nonlinear crystals remain the most appropriate choice.

Since, the parametric gain of SPDC process is low, efforts have been made to improve the brightness of the entangled photon sources by exploring different nonlinear crystals in bulk^8,9^ and waveguide^10^ structures, different phase-matching geometries including type-II^10–15^, type-I^11^ and type-0^15–17^, and different experimental schemes. Given that the length and nonlinearity are two important crystal parameters greatly influencing the overall gain of the SPDC process for a given pump laser intensity, the brightness of the entangled photon sources have been significantly improved over the years through the use of long periodically poled crystals engineered for high effective nonlinearity.

As such, use of type-0, quasi-phase-matching (QPM) in periodically poled potassium titanyl phosphate (PPKTP) crystal in crossed-crystal geometry has produced collinear, non-degenerate paired photons at a spectral brightness as high as 0.278 MHz/mW/nm^17^. On the other hand, PPKTP crystals in type-II phase-matching have been extensively used in Sagnac-loop to produce narrow band entangled photons^18,19^ without exploiting the full advantage of high nonlinear co-efficient of the QPM crystals. Therefore, despite the generation of broadband entangled photons, the type-0 phase-matching is more favored over other phase-matching geometries to produce bright entangled photons. While one can expect further enhancement in the paired photons rate by operating the source at degeneracy, however, the separation and detection of individual photons of the collinear, co-polarized paired photons at degenerate wavelength is significantly difficult proposition. Attempt has been made to overcome such problem with the use of non-collinear, type-0, third order QPM in periodically poled lithium tantalate (PPSLT) crystal^20^, however, the use of third order QPM resulted in moderately low rate of paired photons (98.5 kHz/mW). On the other hand, the requirement of active temperature control for non-critical phase-matching in periodically poled crystals increases the overall complexity of the sources. However, the success of future quantum communication experiments, in addition to other obstacles, demands engineering of high brightness entangled photon sources with minimal system complexities. Here, we demonstrate a high brightness entangled photon source producing non-collinear, degenerate photons at 810 nm with spectral brightness as high as ~0.41 ± 0.02 MHz/mW/nm. Based on a single, 30-mm long PPKTP crystal configured in a polarization Sagnac interferometer^21–25^, the source produces entangled photon states violating the Bell’s inequality by nearly 32 standard deviations and a Bell state fidelity of 0.975. The Table 1 represents the performance of our source as compared to some of the recent bright entangled photon sources based on PPKTP crystals.

**Table 1:** Comparison of the performance parameters of the SPDC sources based on PPKTP crystals.

Reports	Phase-matching	Detected spectral brightness (MHz/mW/nm)	Collection efficiency (%)	Fidelity (%)
ref.^15^	Type-0	0.22^(a)(b)^	31^(c)^	99.1
ref.^16^	Type-0	0.38^(a)(d)^	—	99.3
ref.^17^	Type-0	0.28^(a)(e)^	18^(f)^	98.3
ref.^13^	Type-II	0.002^(c)(g)^	6.6^(c)^	98
Ref.^23^	Type-II	0.005^(a)(b)^	16^(c)^	98.1
Our study	Type-0	0.41^(g)^	18.5^(c)^/20^(f)^	97.5

^(a)^Non-degenerate photons, ^(b)^Sagnac interferometer, ^(c)^single mode fiber, ^(d)^double-pass, ^(e)^two crystals, ^(f)^multimode fiber, ^(g)^single-pass degenerate photons.

## Experiment

**Figure 1:**
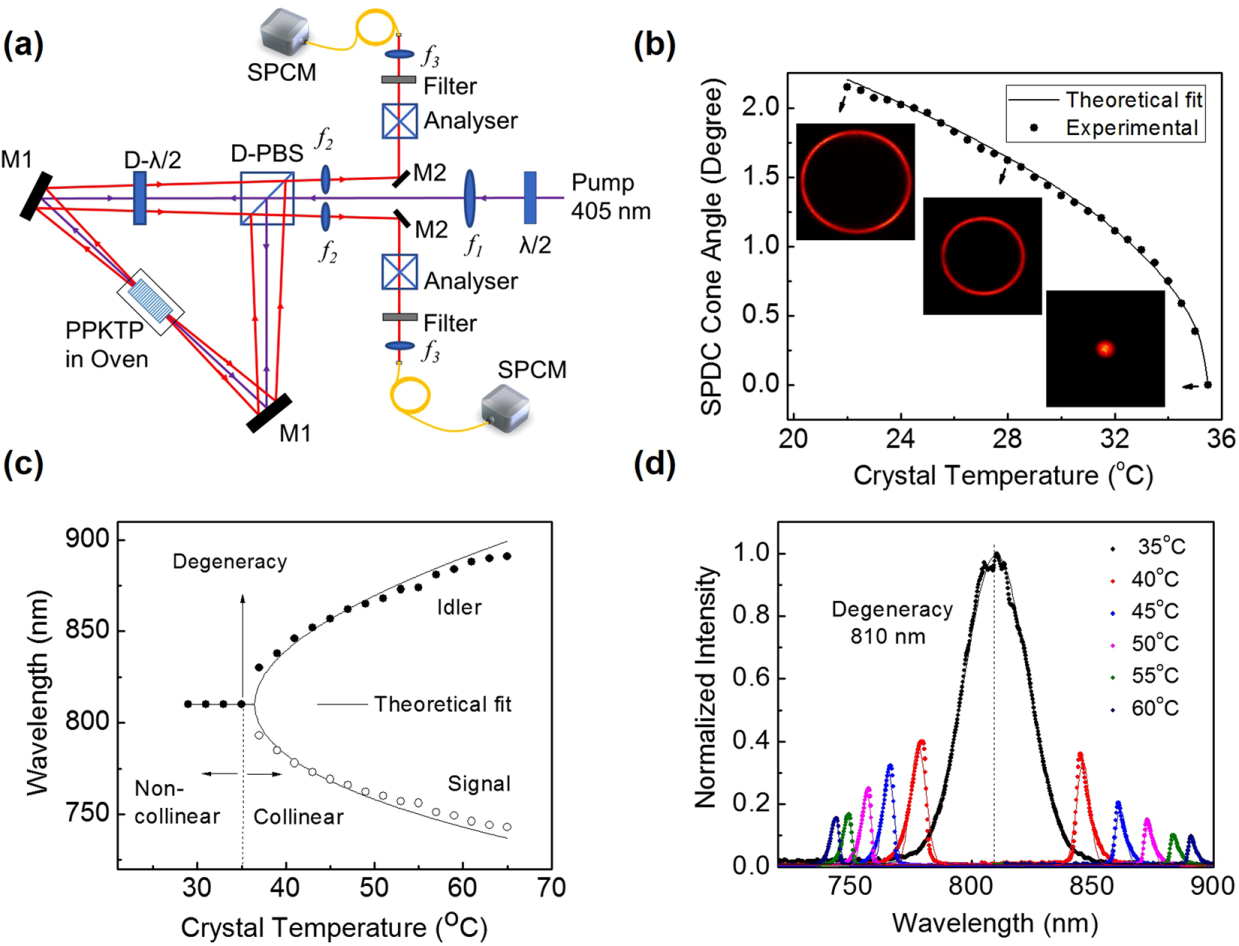
Bright entangled photon source based on degenerate, non-collinear, type-0 phase matching in PPKTP crystal. (**a**) Schematic of the experimental setup. λ/2, half-wave plate at 405 nm; D-PBS, D-λ/2: polarizing beam splitter cube and half-wave plate at dual wavelengths, 405 nm and 810 nm; M1-2, mirrors; *f*1-3, lenses; PPKTP, nonlinear crystal in a temperature oven; Analyzer, polarization analyzer comprises a PBS and a λ/2 plate; IF, interference filter; SPCM, single photon counting module. (**b**) Variation of emission half-cone angle of the SPDC photons measured in free space with crystal temperature. Solid line corresponds to the theoretical fit to the experimental data. (Inset) CCD images of the SPDC ring at three crystal temperatures, 22 °C, 28 °C, and 35 °C. (**c**) Wavelength tuning of the SPDC photons as a function of crystal temperature. (**d**) Emission spectra of the SPDC photons recorded at different crystal temperatures.

The schematic of the experimental setup is shown in Fig. 1(a). A continuous-wave, single-frequency (linewidth <12 MHz) UV laser providing 100 mW of output power at 405 nm is used as a pump laser. A 30 mm long, 2 × 1 mm^2^ in aperture, single grating, PPKTP (Raicol, Israel) crystal of period, Λ = 3.425 µm, is used for type-0 (*e* → *e* + *e*) phase-matched down conversion of pump beam at 405 nm. A convex lens of focal length, *f* = 300 mm, is used to focus the pump beam at the center of the crystal. To generate entangled photons, we have devised a novel experimental scheme based on polarization Sagnac interferometer consisting of a dual wavelength polarizing beam splitter cube (D-PBS), a dual wavelength half-wave plate (D-λ/2) plate, and two high reflecting (R > 99%) mirrors, M, at both 405 nm and 810 nm. The working principle of the scheme can be understood as follows. The λ/2 plate at 405 nm placed before the D-PBS controls the polarization of the pump beam in such a way that the reflection (vertical polarization, V) and transmission (horizontal polarization, H) ports of the D-PBS have equal laser powers. The V polarized pump photons travelling in clock-wise (CW) direction in the Sagnac interferometer, generate V polarized SPDC photons at 810 nm owing to type-0, non-collinear phase-matching in PPKTP crystal. The D-λ/2 plate transforms the polarization of both pump and the SPDC photons from H to V and vice versa. Therefore, the V polarized SPDC photons propagating in CW direction of the Sagnac interferometer transformed into H polarized photons and pass through the D-PBS. At the same time, the H polarized pump photons travelling in the counter clockwise direction (CCW) in the Sagnac interferometer transformed into V polarization after passing through the D-λ/2 plate and generate V polarized non-collinear SPDC photons in the same PPKTP crystal. The D-PBS combines the SPDC photons generated in both CW and CCW directions of Sagnac interferometer. Since both the CW and CCW pump beams follow the same path in opposite directions, and the PPKTP crystal is placed at a position symmetric to the D-PBS, the present experimental scheme is robust against any optical path changes to produce SPDC photons in orthogonal polarizations with ultra-stable phase. Using high reflecting mirrors, M, and two interference filters (IF) of different transmission bandwidths (2 nm and 10 nm) centered at 810 nm, the SPDC photons are collected and subsequently detected with the help of single photon counting module, SPCM  (AQRH-14-FC, Excelitas). The analyzer, comprises a PBS and a λ/2 plate, is used to study the polarization entanglement of the generated photons. The lens pair, *f*_1_ and *f*_2_, images the crystal plane to the fiber tip used for photon collection. For all the measurements presented throughout the manuscript we have used a coincidence window of 8.1 ns.

## Results and Discussions

### Spatial and spectral characteristics of the SPDC source

To verify the generation of degenerate SPDC photons in non-collinear, type-0 phase matching, we pumped the crystal at an input power of 40 mW. Using a CCD camera (SP620U, Spiricon) along with an interference filter of spectral width ~10 nm centered at 810 nm, we have recorded the angular spectrum or transverse momentum distribution of the SPDC photons in the Fourier plane using a convex lens of focal length, *f* = 50 mm in *f*-*f* optical system configuration^26^. Since each pixel represents a particular transverse momentum of the SPDC photons, using the recorded spatial distribution of the SPDC photons we calculated the emission angle (half cone angle) of the photons at different crystal temperatures with the results shown in Fig. 1(b). As evident from Fig. 1(b), the emission angle of the SPDC photons decreases from 2.15° to 0° with the increase of crystal temperature from 22 °C to 35.5 °C, clearly verifying the transition of the phase-matching of the SPDC photons from non-collinear to collinear geometry. While decrease in crystal temperature below 22 °C results in further increase in the non-collinear phase-matching angle of the degenerate SPDC photons, the increase of crystal temperature beyond 35° produces non-degenerate SPDC photons in collinear phase-matching geometry. A linear fit (not shown in the Fig. 1(b)) to the experimental data for the crystal temperature variation across 22 °C to 29 °C reveals that the emission angle (half cone angle) of the SPDC photons changes by 0.037° for a change of 1 °C in the crystal temperature. Such a small variation in the emission angle with the crystal temperature indicates the possibility of generation of entangled photons at room temperature without using any active temperature control, commonly required for non-critical phase-matching^27^. Using the Sellmeier equations^28^ of PPKTP crystals and the equations for non-collinear phase-matching we have theoretically calculated (solid line) the emission angle of the SPDC photons in good agreement with the experimental data (dots). The non-collinear generation and variation of the SPDC ring diameter with crystal temperature is also evident from the CCD images of the intensity distribution of the SPDC photons as shown by the inset of Fig. 1(b). In contrary to the electron multiplying CCD (EMCCD) commonly used to record SPDC rings, here, the use of CCD camera signifies the generation of high number of SPDC paired photons in our experiment. We have also studied the spectral distribution of the SPDC source at both non-collinear and collinear phase-matching geometries. Pumping the crystal with an input power of 40 mW and removing the interference filter we have measured the spectrum of the SPDC photons using a spectrometer (HR 4000, Ocean Optics) at different temperatures of the crystal. The results are shown in Fig. 1(c). As evident from Fig. 1(c), for the crystal temperature between 29 °C to 35 °C results in non-collinear, degenerate SPDC photons at 810 nm, however, further increase in the crystal temperature up to 65 °C produces collinear, non-degenerate SPDC photons with wavelength tunability over 810–743 nm in the signal wavelength (open circles) and 810–891 nm in the idler (solid circle). The solid line is the tuning curve predicted from the Sellmeier equations of PPKTP crystal^28^, confirming a reasonable agreement between the experimental data and theoretical calculations. Figure 1(d) shows the measured spectra of the collinear SPDC paired photons at crystal temperature of 35°, 40°, 45°, 50°, 55°, and 60 °C. It is evident from Fig. 1(d) that the source produces degenerate SPDC photons at a spectral width (full width at half maximum, FWHM) of ~31 nm centered at 810 nm, with a signature of high parametric gain of the PPKTP crystal. We also observed similar spectrum at crystal temperature below 35 °C. However, the spectral width of the signal and idler photons decrease with the increase of crystal temperature away from degeneracy. From the normalized intensity of the SPDC photons, it is also evident that the development of SPDC source with high brightness requires operation of the periodically poled crystal based sources in degenerate, non-collinear, type-0 phase matching geometry.

### Detection and characterization of the SPDC photons using both multimode and single mode fibers

**Figure 2:**
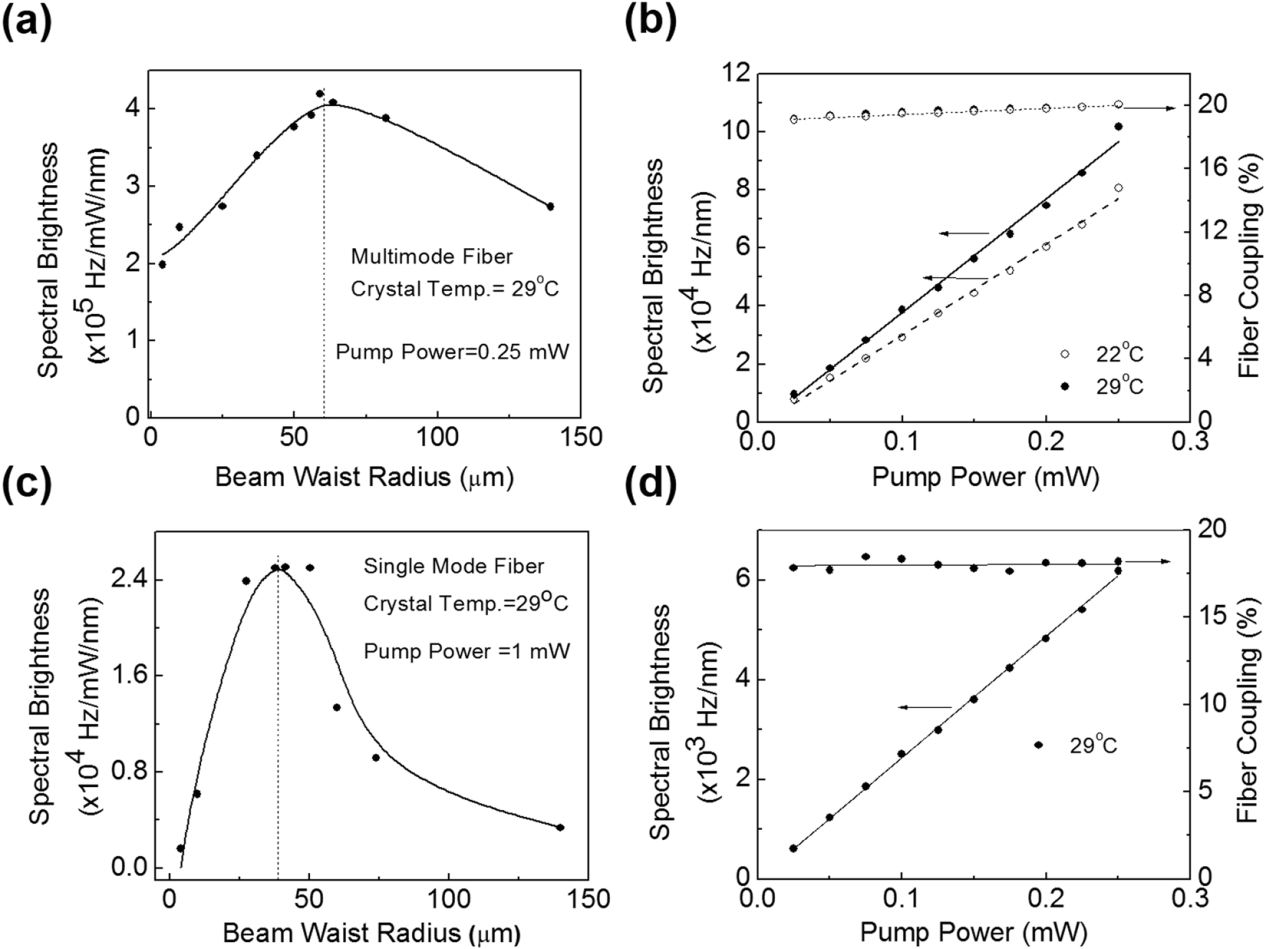
Characteristics of the degenerate, non-collinear SPDC source. (**a**) Dependence of spectral brightness of the paired photons on the pump beam waist at crystal temperature of 29 °C, and (**b**) the variation of spectral brightness and fiber coupling efficiency on the pump power at crystal temperature 22 °C (open circles) and 29 °C (solid circles) for photon collection using multimode fiber. Similarly, for photon collection using single mode fiber, (**c**) dependence of spectral brightness of the paired photons on the pump beam waist, and (**b**) the variation of spectral brightness and fiber coupling efficiency with the pump power at crystal temperature of 29 °C. Lines are guide to the eye.

After successful generation of degenerate, non-collinear SPDC photons using type-0 phase-matched PPKTP crystal, we have  characterized the source in terms of spectral brightness and efficiency. In order to collect photons from two diametrically opposite points of the SPDC ring we have used the imaging systems consist of two convex lenses of focal lengths, *f*_1_ and *f*_2_, in 2*f*_1_-2*f*_2_ configuration to image the crystal plane to the tip of the fiber connected to SPCM. For comparison, we have used both multimode(MMF) and single mode fibers(SMF) separately to measure the spectral brightness of the photons collected from two diametrically opposite points on the SPDC ring filtered with the interference filter (IF) of bandwidth ~2 nm at different pump beam focusing and pump power. The results are shown in Fig. 2. As evident from Fig. 2(a), for a constant pump beam power of 0.25 mW and crystal temperature of 29 °C the spectral brightness of the paired photons, collected using multimode fiber and the imaging system having lenses of focal lengths, *f*_1_ = 50 mm and *f*_2_ = 5 mm, vary from 1.98 ± 0.02 × 10^5^ Hz/mW/nm to 2.73 ± 0.02 × 10^5^ Hz/mW/nm with the pump beam waist radius varying from 4 µm to 139.5 µm clearly showing a maximum spectral brightness of 4.19 ± 0.03 × 10^5^ Hz/mW/nm at optimum pump beam waist radius of 59 µm. We have also measured the dependence of the spectral brightness with the pump power at two different crystal temperatures, 22 °C and 29 °C. The results are shown in Fig. 2(b). As expected, the spectral brightness increases linear to the pump power (see Fig. 2(b)), at a slope of, Nc = 3.13 ± 0.01 × 10^5^ Hz/mW/nm, and Nc = 3.92 ± 0.01 × 10^5^ Hz/mW/nm at crystal temperature 22 °C (open circle) and 29 °C (closed circle) respectively. Using the photon numbers, N1~N2 recorded from two diametrically opposite points on the SPDC ring and their spectral brightness, Nc in the equation, N_Pair_ = (N1 × N2)/Nc^20^, we can estimate the spectral brightness to be as high as ~8.05 ± 0.03 MHz/mW/nm and ~10.16 ± 0.02 MHz/mW/nm at 22 °C and 29 °C respectively. Such a high number of non-collinear degenerate paired photons as compared to previous reports, can be attributed to high parametric gain resulting from the use of long crystal length and first order grating period of the PPKTP crystal. To the best of our knowledge, this is so far, the highest detected non-collinear, degenerate paired photons from a bulk crystal based SPDC source. Given that the parametric gain is almost constant near the degeneracy and the SPDC is a very feeble nonlinear process, one can, in principle, expect the number of detected paired photons to be constant with crystal temperature. However, as evident from Fig. 2(b), we observed a change in spectral brightness with crystal temperature. Such effect can be understood as follows. Given that the number of SPDC photons is constant at constant pump power, and distributed over the annular area of the SPDC ring, the linear decrease in the radius of the SPDC ring with the increase in crystal temperature from 22 °C to 29 °C results in a quadratic increase in the number density (number of photons per unit area per sec). Therefore, the spectral brightness measured using a  fixed aperture size of the detection system show the change with the crystal temperature. Here, we can conclude that for high brightness SPDC source one need to adjust the crystal parameters to access the optimum size of the SPDC ring. One can in principle, adjust the grating period of the PPKTP crystal to access such high brightness of the SPDC source at any favorable crystal temperature. The coupling efficiency calculated using the equation, $\xi =Nc/\sqrt{N1\ast N1}$ ${\boldsymbol{,}}$ for different pump powers, as shown in Fig. 2(b), remains almost constant in the range of 19% to 20% at both crystal temperatures. Using the same experimental condition, we have verified the room temperature operation of the source by measuring the coincidence and singles counts without using temperature oven. At the lab temperature of 23 °C, we observed the spectral brightness of the source to vary with a standard deviation as low as ~1.6% over two hours.

Further, we have characterized the SPDC source using single mode fibers. Pumping the crystal at a constant power of 0.25 mW and temperature ~29 °C we have measured the photon spectral brightness collected using the single mode fiber and the imaging systems comprise lenses of *f*_1_ = 100 mm and *f*_2_ = 2 mm for different pump beam waist radius. As evident from Fig. 2(c), the spectral brightness varies from 0.164 ± 0.004 × 10^4^ Hz/mW/nm to 0.336 ± 0.006 × 10^4^ Hz/mW/nm with the pump beam waist radius varying from 4 µm to 139.5 µm clearly showing a maximum spectral brightness of 2.50 ± 0.02 × 10^4^ Hz/mW/nm at optimum pump beam waist radius of 41 µm. Keeping the pump beam waist radius at optimum value of 41 µm, we have measured the variation of spectral brightness with the pump power. As evident from Fig. 2(d), the number of paired photons (spectral brightness) increases linear to the pump power at a slope of Nc = 2.43 ± 0.01 × 10^4^ Hz/mW/nm resulting a maximum paired photons of 6.18 ± 0.01 × 10^3^ Hz/nm at a pump power of 0.25 mW. The corresponding maximum number of paired photons produced in the SPDC process can be calculated to be N_Pair_ = 0.74 ± 0.01 MHz/mW/nm. The coupling efficiency is almost constant at ~18.45% for all the pump powers.

### Entanglement properties of the source

**Figure 3:**
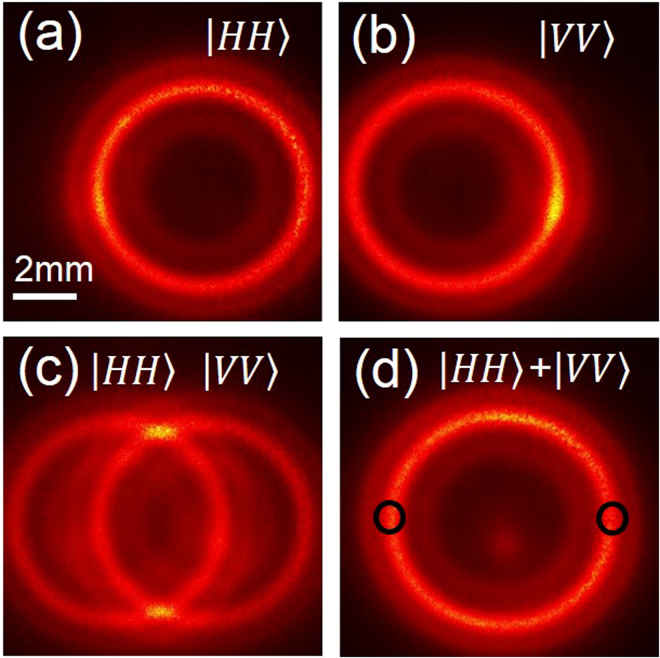
Spatial distribution of the SPDC paired photon recorded using EMCCD. Images of the paired photons of polarization state, (**a**) $|HH\rangle$. (**b**) $|VV\rangle$ (**c**) both $|HH\rangle$ and $|VV\rangle$ with misalignment, and (**d**) perfectly overlapped states, $|HH\rangle$ and $|VV\rangle$ The black circles represent the diametrically opposite regions on the SPDC ring used for entanglement studies. The pump power used for this measurement is 5 mW.

With the successful generation of paired photons with high brightness, we study the source for the generation of polarized entangled photons. Since in type-0 phase-matching geometry, the generated paired photons have the same state of polarization as that of the pump photons, we placed the PPKTP crystal along with a D-λ/2 plate inside the polarization Sagnac interferometer and pumped in both CW and CCW directions with same state of polarization (see Fig. 1(a)), vertical (|V〉). The non-collinear SPDC photons generated in both the directions are superposed using the PBS after changing the polarization state of the photons generated in one of the directions by the D-λ/2. To verify the non-collinear generation of SPDC photons for both pump directions, and their superposition as required for entanglement, we have recorded the intensity distribution of the SPDC photons using an EMCCD (ANDOR, DU-897U-CS0-BVAntiF) at pump power of 5 mW. The results are shown in Fig. 3. Adjusting the polarization state, horizontal (|H〉), and or vertical (|V〉), of the pump beam to the PBS, we pumped the PPKTP crystal in CCW and or CW directions, respectively. Figure 3(a) and (b) show the spatial distribution of the SPDC photon with polarization states, |HH〉 and |VV〉 generated in CW and CCW, respectively. It is evident that the PPKTP crystal in the current experimental architecture produces indistinguishable SPDC rings of orthogonal polarization states, |HH〉 and |VV〉, which is further confirmed from the Fig. 3(c) and (d) representing the images of the SPDC rings under misalignment and perfect alignment of the source, respectively. Under perfect alignment, the twin photon states can expected to be, $\Psi =\frac{1}{\sqrt{2}}(|HH+{e}^{i\phi }|VV)$. However, one need quantum state tomography study to ascertain the actual twin photon state.

**Figure 4:**
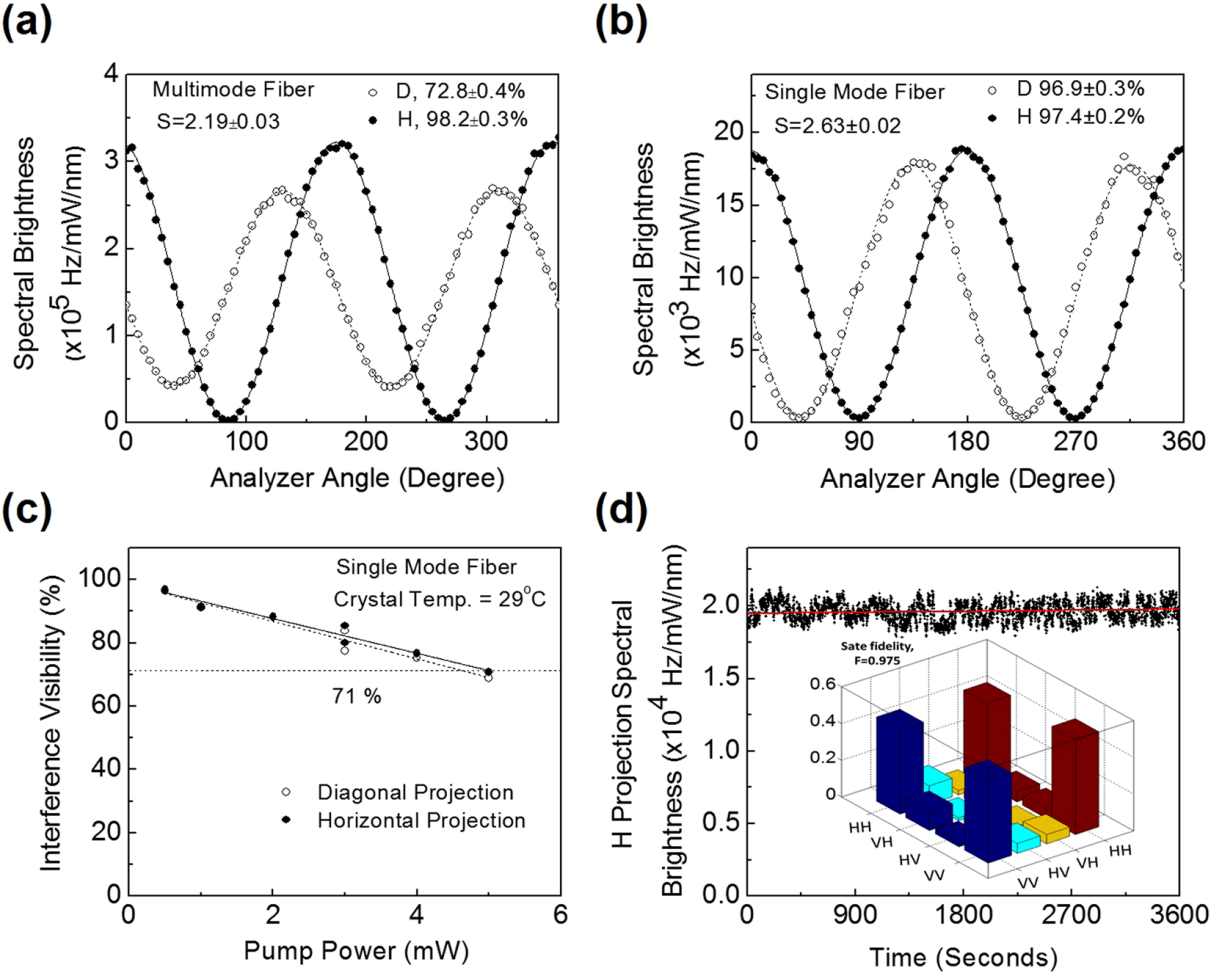
Entanglement characteristics of the SPDC source. Quantum interference of the entangled photons at horizontal, H (solid circles) and diagonal, D (open circles) projection bases for crystal temperature 29 °C collected using (**a**) multimode fiber, and (**b**) single mode fiber. (**c**) Dependence of interference visibility of the entangled photon states, projected in H (solid circles) and D (open circles) bases, on the input pump power. (**d**) Stability of the spectral brightness of the paired photon recorded over a time period of 3600 seconds with an exposure of 1 second. (Inset) Graphical representation of the absolute values of the density matrix of the polarization entangled photon states.

For quantitative analysis of the polarized entanglement of the source, we pumped the crystal with a total power of ~1 mW at crystal temperature ~29 °C and aligned the system to maintain perfect overlapping of the SPDC rings. Using standard coincidence measurement technique we recorded the two-photon interference in terms of photon coincidence between the twin photons distributed in two diametrically opposite points of the SPDC ring under two non-orthogonal projection bases, H/V (horizontal/vertical) and D/A (diagonal/anti-diagonal). The photon detection system comprised with two lenses of focal length, *f*_1_ = 150 mm and *f*_2_ = 5 mm in 2*f*_1_-2*f*_2_ configuration, an interference filter of bandwidth ~2 nm, polarization analyser, multimode fiber, and SPCM. The results are shown in Fig. 4. As evident from Fig. 4(a), we observed a typical quantum interference of the polarized entangled photons for H/V (solid cicles) and D/A (open circles) projections for the crystal temperature 29 °C. The solid lines are best fit to the experimental data. The number of detected entangled paired photons has a maximum value of 0.32 ± 0.02 MHz/mW/nm, close to the detected paired photons (Nc = 3.92 ± 0.01 × 10^5^ Hz/mW/nm) presented in Fig. 2(a). Such small difference can be attributed to the use of longer focal length, *f*_1_ = 150 mm, of the detection system to avoid mechanical constraint in the experimental setup.

The interference fringe visibility in H and D projections are estimated to be 98.2 ± 0.3% and 72.8 ± 0.4% respectively. The measured fringe visibility under both bases are higher than 71%, sufficient to violate Bell’s inequality. Using the coincidence rates, we measured the Bell’s parameter to be S = 2.19 ± 0.03, violating the Bell’s inequality by nearly 6 standard deviations, a clear indication of polarization entanglement of the generated two-photon states. The lower value of fringe visibility in diagonal projection can be attributed to the relative phase among the higher order spatial modes in the SPDC process collected by the multimode fiber, which can be improved through proper phase compensation schemes^29^. Similarly, we have studied the polarization entanglement using single mode fiber with the results shown in Fig. 4(b). As evident from Fig. 4(b), the number of detected entangled paired photons has a maximum value of 1.88 × 10^4^ Hz/mW/nm at 0.5 mW of pump power. However, the interference fringe visibility in H (solid circles) and D (open circles) projections are measured to be 97.4 ± 0.2% and 96.9 ± 0.3%. Using the coincidence counts we have calculated the Bell’s parameter to be S = 2.63 ± 0.02, clearly violating the Bell’s inequality by nearly 32 standard deviations. Such study clearly shows the generation of high quality entangled photons with high brightness even in the absence of any phase compensation schemes, as commonly used for SPDC based entangled photon sources. The Table 2 summaries various experimental results of the SPDC source.

**Table 2:** Performance parameters of the SPDC source measured using both single mode and multimode fibers at the crystal temperature of 29 °C.

Collection Fiber	Detected spectral brightness (MHz/mW/nm)	Estimated spectral brightness (MHz/mW/nm)	Coupling efficiency (%)	Visibility (%) H/D bases	Fidelity (%)
Single mode	0.024	0.74	18.5	97.4 ± 0.2/96.9 ± 0.3	97.5
Multimode	0.41	10.16	20	98.2 ± 0.3/72.8 ± 0.4	—

We have also studied the effect of pump power on the entanglement quality of the paired photons by measuring the visibility of quantum interference fringes at different pump powers with the results shown in Fig. 4(c). Like previous report^12^, here, we have also observed that the fringe visibility in both H and D projections decrease with the pump power resulting a fringe visibility of 70.8% and 68.9% in H and D projections respectively, below the fringe visibility limit of 71% for entanglement, even for the pump power as low as 5 mW. Such degradation in the entanglement quality with the increase of pump power can be attributed to the multi-pair generation due to high gain in the PPKTP crystal. To verify the robustness of our entangled photon source, we have measured the fluctuation in the spectral brightness at H-H projection collected using single mode fiber. Keeping the pump power constant at 0.5 mW, we have recorded the coincidence of the paired photons with an exposure of one second over 3600 seconds with the results shown in Fig. 4(d). As evident from Fig. 4(d), the spectral brightness of the SPDC source fluctuate at a standard deviation of 0.065 × 10^4^ Hz/nm over the average spectral brightness of 1.97 × 10^4^ Hz/nm for 0.5 mW, corresponding to an estimated passive fluctuation as low as ~3%. Similarly, in D-A projection we have measured the fluctuation in spectral brightness at a standard deviation of 0.074 × 10^4^ Hz/nm with an estimated passive fluctuation of ~4.7%, slightly higher than that of H-H projection. Such instability of the SPDC source can further be improved with proper isolation of the source from the temperature fluctuation and air turbulence of the laboratory environment. To find the degree of entanglement of the photon states we have constructed the density matrix of the state using linear tomographic technique^30^. The inset of Fig. 4(d) shows the graphical representation of the absolute values of the density matrix of the generated states. From the analysis, we determine the state to be, $\Psi =\frac{1}{\sqrt{2}}(|HH\rangle -|VV\rangle )$ with state fidelity of 0.975. Since the relative phase between the CW and CCW beams of the Sagnac interferometer is determined by the position of the crystal^25^, we can transform the output state, $\Psi =\frac{1}{\sqrt{2}}(|HH\rangle -|VV\rangle )$ into $\Psi =\frac{1}{\sqrt{2}}(|HH\rangle +|VV\rangle )$ and vice versa by simply adjusting the crystal position with respect to the PBS in our experimental setup.

In conclusion, we have demonstrated a simple, compact and robust source of entangled photons at high brightness. Based on non-collinear, degenerate, type-0 phase-matched single PPKTP crystal at room temperature in a Sagnac interferometer, the source produces a detected paired photons rate of 0.41 ± 0.02 MHz/mW/nm. To the best of our knowledge, this is the highest number of degenerate paired photons detected with the help of multimode fiber from a bulk crystal pumped with a continuous-wave laser. The polarization correlation study and quantum tomography measurement using single mode fibers reveals that the source produces entangled photon states violating the Bell’s inequality by nearly 36 standard deviations and a Bell state fidelity of 0.975. Such high brightness entangled photon source in compact and rugged architecture is ideal for the many present and future experiments in the field of quantum optics especially in quantum communications.
